# Social Integration and Health Among Young Migrants in China: Mediated by Social Mentality and Moderated by Gender

**DOI:** 10.3389/fpsyg.2022.863443

**Published:** 2022-04-25

**Authors:** Jingjing Zhou, Li Zhu, Junwei Zhang

**Affiliations:** ^1^School of Sociology and Population Studies, Nanjing University of Posts and Telecommunications, Nanjing, China; ^2^Department of Sociology, School of Social and Behavioral Sciences, Nanjing University, Nanjing, China; ^3^College of Philosophy, Law and Political Science, Shanghai Normal University, Shanghai, China

**Keywords:** social integration, social mentality, health status, young migrants, China

## Abstract

Population mobility has been one of the most basic social characteristics of China’s reform and opening up for more than 40 years. As the main labor force in Chinese cities, young migrants have made major contributions toward China’s economic miracle as the country has experienced rapid industrialization and urbanization. However, frequent mobility has caused an imbalanced social mentality in young migrants and often leads to issues with social integration, which has made this group more vulnerable with respect to their health. This study used the 2013 and 2015 Chinese General Social Survey (CGSS) data of 1,007 young migrants to investigate social mentality mediating the linkage between social integration and health among young migrants. Additionally, to probe the moderating role of gender, multi-group structural equation modeling was applied to test if the pathways in the mediation model differed between young male and female migrants in a significant way. The results suggested that after controlling for the influence of age, marital status, education, and personal annual income, social integration positively impacted the health of young migrants in a significant way; social mentality mediated the action of social integration on this group’s health; and with respect to gender difference, on the three paths of social integration affecting health, social integration affecting social mentality, and social mentality affecting health, young male migrants were more affected than young female migrants. The findings of this study could help improve gender-specific policies on the health of the floating population and offer important theoretical reference and practical suggestions for future research.

## Introduction

With the acceleration of industrialization and urbanization, large-scale urban–rural population mobility has become one of the fundamental social characteristics of China’s reform and opening up (Unger and Siu, 2019). According to the seventh national census, China’s internal migrants numbered 376 million in 2020, accounting for one-fourth of the total population, of which 73.6% were under 40 years old (Cheng and Duan, 2021; National Bureau of Statistics of China, 2021). A growing number of evidence indicate that frequent mobility and the traumatic events that it brings lead to health attrition among young migrants (Mwanri and Mude, 2021). The expedited economic development in China has deepened inequalities in the society, causing a decline in social trust and public confidence about the future (Su et al., 2019). Furthermore, China’s floating population is undergoing a generational transition. Although young migrants, also known as new-generation migrants, are more open to and capable of social integration (He and Wang, 2016), they face stronger social pressures and are more likely to ignore their own health problems. Consequently, their quality of life and health are more vulnerable than those of older-generation migrants (Yu et al., 2019; Zeng et al., 2020). In addition, the COVID-19 pandemic has not only increased health risks, but also contributed to negative social emotions (Elisabeth et al., 2020). Under such circumstances, particular attention, therefore, must be paid to the improvement of the health status among young migrants.

Generally speaking, socioeconomic status, environment for working and living, medical resources, and lifestyle are regarded as the main social factors that affect population health (Rechel et al., 2013). However, for young migrants, frequent migration exposes them to more social integration problems, so the degree of social integration has stronger explanatory power over their health status (Fan et al., 2020). Relevant empirical studies hold that the extent of social integration significantly affects the health status and quality of life among the migrants (Lin et al., 2017). Moreover, the social integration of young migrants is gradual, interactive, and continual, and the personal feelings and experiences that this group encounters during the process of flow and integration are embedded in the social transformation of China, which affects their social mentality (Zhou, 2014). Due to the diffusive and dynamic nature of social mentality, its effect on health has been well documented (Xi et al., 2021). Social integration can improve mental health by enhancing self-control, sense of belonging, and generalized trust, which provides empirical possibilities for social mentality mediating the interaction between social integration and health for young migrants (Na and Hample, 2016).

In addition, there is growing evidence that gender differences are significant among young migrants with respect to social integration, social mentality, and health (Klein et al., 2020; Starck et al., 2020), which are often masked by complexities of migrant backgrounds and migration processes (Wandschneider et al., 2020) and vary across cultures and countries (Becchetti and Conzo, 2022). Besides, relevant studies have paid less attention to the relationships among social integration, social mentality, and health, as well as gender differences among young migrants in the Chinese context. In China, rapid economic development has led to a deepening of social inequality, which can easily lead to an imbalance in the social mentality of the floating population (Evans and Rubin, 2021). Greater social pressure and faster pace of life not only complicate the social integration of young migrants, but also pose a greater challenge to their health (Wu et al., 2018). Therefore, using the Chinese General Social Survey (CGSS) data in 2013 and 2015, this study explored the mechanisms of interaction that exist among young migrants’ social integration, social mentality, and health. It also examined the mediating effect of social mentality in linking social integration and health of young migrants. Multi-group verification was conducted on gender differences in the above-mentioned mechanisms in order to offer suggestions for addressing gender inequalities in policies related to the health of young migrants.

## Literature Review

### Social Integration and Health

Social integration is multi-dimensional. Although existing studies are divided, most of them include dimensions, such as economy, culture, social interaction, and psychological identity (Yang, 2015). Many empirical studies have shown that social integration can significantly improve self-rated health (Ma and Xia, 2021) and mental health (Chen et al., 2019) and reduce the probability of disease (Freak-Poli et al., 2021) in the floating population. However, although social integration positively affects the health and QOL of migrants, the underlying mechanism therein has yet to be fully discussed. Some scholars have pointed out that the extent to which social integration affects health depends on the local environment of a specific destination and that future research needs to focus more on differences in social integration processes rather than differences in outcomes (Akresh et al., 2016).

Existing studies have proposed several possible explanatory mechanisms. Some scholars believe that social integration helps migrants build social networks in inflow areas and improves their health by increasing access to informal sources of social support (Holt-Lunstad, 2015). From the perspective of resource acquisition, some scholars believe that social integration can help the floating population access and utilize medical and health information and resources in the inflow area and promote the improvement of migrants’ health (Liang et al., 2020). However, increased social integration does not necessarily lead to improved health. A Korean study showed that in some cases, social disorganization actually had a positive effect on individual health (Jo et al., 2020).

### Social Mentality as Mediator

Social mentality can be defined as the sum of socio-psychological conditions that people generally experience in a certain area in specific social, historical, and cultural contexts (Yang, 2019). In the context of migration, a positive social mindset can attenuate the negative effects of migration and integration stress on health outcomes (Jiang et al., 2019; Yang et al., 2019). Some studies have also demonstrated the positive impact of social integration on social mentality, such as social justice, social trust, and subjective wellbeing (Appau et al., 2019; Yang and Wang, 2021). These studies have laid the foundation for empirical research of the mediating effect of social mentality on the association between social integration and health.

In addition, some scholars have used the social integration theory and the attachment theory to discuss the pathways from social integration to health, including behavioral pathways, psychological pathways, and physiological pathways (Berkman et al., 2000), which offer theoretical possibilities for the mediation of social mentality between social integration and health. At present, the psychological pathways that social integration indirectly affects health through individual psychological factors, such as self-esteem and sense of belonging, have been verified extensively (Schwager et al., 2020; Chen et al., 2020b). A small body of research has discussed the specific dimensions of social mentality, such as social trust and subjective wellbeing, as mediating factors between social integration and health (Steel et al., 2018; Zhang et al., 2021). However, there is a lack of complete and clear-cut research and empirical explanation for the mechanism of action among young migrants’ social integration, social mentality, and health.

### Gender Differences

Gender differences are widely discussed in studies of young migrants’ social integration, social mentality, and health (Kamis and Copeland, 2020; Park et al., 2021). Existing studies have demonstrated that a consensus has yet to be reached about how gender differences impact the health of young migrants across national contexts. More discussion is thus warranted from the perspective of social structure and socio-cultural aspects (Cheng et al., 2021). For example, a Korean study argued that under totalitarian and patriarchal cultures, men’s subjective social class and wellbeing are more likely to be mediated by the quality of social relations (Kim et al., 2020). In Finland, which is a more socially tolerant country, women are better positioned to establish positive social relations and gain social trust than men (Grigaityte et al., 2020).

Existing literature in China argues that better social integration among young migrants can improve their health status, but the self-rated health of women is better than that of men (Lin et al., 2016) due to the fact that men experience more social pressure (Li et al., 2018). Some scholars have indicated that young female migrants receive more social support from social networks in the inflow area, which helps improve their mental health (Wang, 2021). However, young female migrants are less socially integrated than males, which may be linked with the fact that they experience higher rates of unemployment because of their family care responsibilities (Tong and Kawachi, 2020).

### Insufficiencies in the Current Literature and Hypotheses Establishment

**Figure 1:**
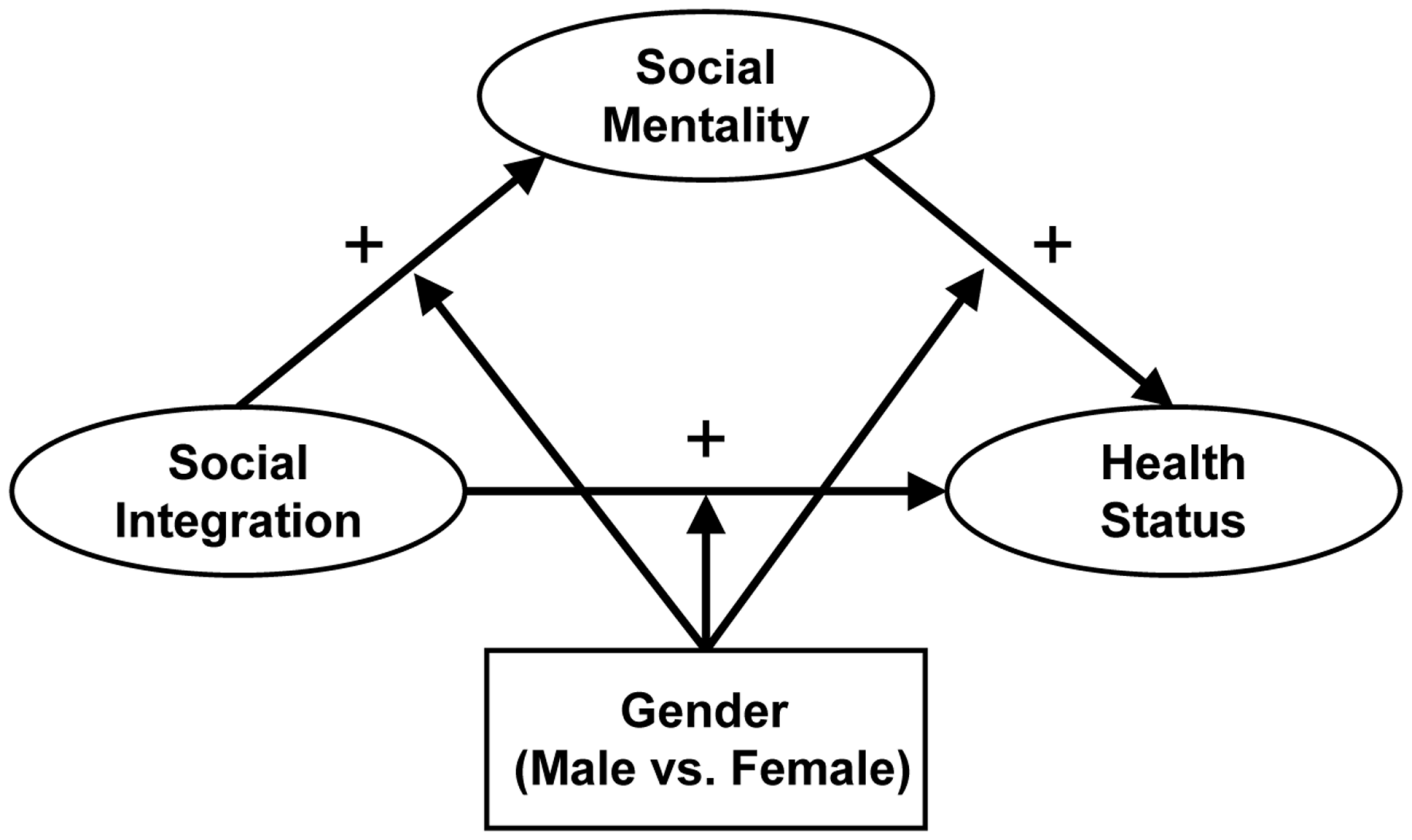
Conceptual framework.

Most of the existing research has looked into the direct impact of young migrants’ social integration and social mentality on their health (Campostrini et al., 2019) or explained how social integration affects social mentality (Xiao et al., 2022). The mechanisms of interaction that exist among young migrants’ social integration, social mentality, and health are, however, partially or implicitly explained. Moreover, there is a lack of attention to gender difference in the way social mentality mediates the interaction between social integration and health among young migrants, and the difference along gender lines in the intensity of social integration and social mentality affecting health is rarely tested in a comprehensive manner. Therefore, a comprehensive conceptual framework was established herein to explain the relationships among social integration, social mentality, and health status as well as gender differences among young migrants in China (see Figure 1). Three hypotheses were proposed in the study:

*H1:* The higher the degree of social integration, the better the health status for young migrants.

*H2:* Increased social integration may improve the social mentality of young migrants, thereby improving their health status.

*H3:* How social integration acts on the health of young migrants through social mentality may differ by gender.

## Materials and Methods

### Data

The data used in this study were derived from the Chinese General Social Survey. It is a large-scale comprehensive social survey designed and implemented by the National Survey Research Center at Renmin University of China. Multi-stage random probability sampling is used to cover 28 provinces, autonomous regions, and municipalities in China (excluding Hainan, Xinjiang, Tibet and Hong Kong, Macao, and Taiwan regions). The surveyed content involves many aspects of the respondents, such as background information, marital and family status, work and income, attitude, and behaviors, indicating a strong representativeness and credibility (Wang and Tang, 2012).

Questions to measure the variables of this research can be found in the questionnaires of CGSS2013 and CGSS2015. Unfortunately, questions regarding the economic integration, which is one of the dimensions of social integration of young migrants, do not appear in the latest data released in CGSS2018. Since our analysis herein focuses on the mechanism of action between social integration, social mentality, and health, CGSS2013 and CGSS2015 offer ideal data for our research purpose. Using the data of CGSS2013 and CGSS2015, we built a pooled cross-section dataset with a total sample size of 21,692 individuals. Because the object of study is young migrants, respondents aged between 15 and 44 years (Settles and Steinmetz, 2013) with registered residences outside of the district/county/county-level city in question (Xu and Chen, 2020) were retained, and after removing missing values, the sample that entered the analysis consisted of 1,007 young migrants.

### Measurement

#### Health Status

In studies measuring health status, scholars usually use the indicator of self-rated health. Compared with other indicators, self-rated health has better stability (Li et al., 2019), and even when objective health conditions are considered, self-rated health can still better represent individual health status (Idler and Benyamini, 1997). This study took subjective health assessments from the CGSS questionnaire to measure “health status.” With reference to the concept of multidimensional health (Wolinsky, 1980), the question “How often have health issues impacted your work among other daily activities in the past month?” was asked to assess whether health affected life, and the question “How often have you experienced upset or depression in the past month?” was asked to determine their mental health. Response options to both questions were divided on a five-point scale (1 = always, 5 = never).

#### Social Integration

To measure social integration, most studies divide it into economic integration, cultural integration or acculturation, identity integration, and psychological integration (Entzinger, 2006). Such division may vary in denotations, but implies similar connotation and measures (Daiute et al., 2021). This study intended to divide social integration into three dimensions: economic integration, acculturation, and social adaptation. Among them, economic integration refers to the integration of young migrants with regard to employment, income, and social security (Wu et al., 2019). Subjective social status was assessed based on responses to the following question from the CGSS questionnaire: “What do you think is your socioeconomic status compared with peers?” (1 = low, 2 = moderate, 3 = high). Acculturation refers to the familiarity of young migrants with the language, lifestyle, and social customs of the inflow area and the extent of their participation in cultural activities (Zhou, 2012). Questions like “How often have you taken part in cultural activities when you are free, such as concerts, performances, and exhibitions, in the past year?” were asked to measure one’s frequency of participation in cultural activities. The responses ranged across five values with a higher value indicating a higher frequency (1 = never, 5 = every day). Social adaptation means the reciprocal acceptance between young migrants and local residents during social interactions (Gan and Zhang, 2021). The following questions were asked to assess social adaptation: “How often do you engage in social and recreational activities with neighbors (such as dropping by each other’s home, watching TV, eating, and playing cards?)” and “How often do you engage in social and recreational activities with friends (dropping by each other’s home, watching TV, eating, playing cards, etc.)?” Responses to both questions were divided into seven options with assigned values in which the higher the value, the higher the frequency (1 = never, 7 = almost every day).

#### Social Mentality

Social mentality can be defined as the sum of perceptions, emotions, values, behavioral intentions, and social cognition existing within a certain social group for a certain period of time, which is related to specific social operating conditions or major social changes (Liu et al., 2018). Most scholars take the measures of social mentality, such as emotions, values, behavioral intentions, and social cognition (Lutzman et al., 2021). This study measured the social mentality of young Chinese migrants based on social trust, sense of social equality, and subjective wellbeing (Wang, 2018). With respect to generalized social trust, the CGSS asked the question “In general, do you agree that the vast majority of members in this society are trustworthy?” (1 = strongly disagree, 5 = strongly agree). To assess social equality, the respondents were asked, “In general, how fair do you think today’s society is?” (1 = completely unfair, 5 = completely fair). To determine subjective wellbeing, the respondents were asked, “Generally speaking, how happy are you with your life?” (1 = very unhappy, 5 = very happy).

#### Covariates

Covariates in this study consisted of demographic characteristics of young migrants, such as gender (0 = male, 1 = female), marital status [single (0 = no, 1 = yes), married (0 = no, 1 = yes), divorced (0 = no, 1 = yes)], personal annual income divided into five categories ranging from <20,000 CNY to ≥80,000 CNY, age, and years of education.

### Analytical Approach

This study used SPSS 23.0 to organize data, generate descriptive statistics, and run correlation analyses. In line with the purpose of this study, structural equation modeling (SEM) was carried out among the variables in Amos24.0 in three steps. First, the confirmatory factor analysis (CFA) was employed to test the measurement model that contained three latent variables, namely, the health status, social integration, and social mentality of young migrants. This study used chi-square (*χ*^2^), comparative fit index (CFI), Tucker–Lewis index (TLI), root mean square of approximation error (RMSEA), and standardized residual root mean square (SRMR) to assess the goodness of fit with non-significant chi-square values (*p* > 0.05), CFI and TLI values above 0.90, and RMSEA and SRMR below 0.08 indicating a good model-data fit (Siedlecki et al., 2014). Second, a structural equation model containing the mediating valuable—young migrants’ social mentality—was tested. Bootstrapping analysis was done to verify how significant the mediation effect was (5,000 re-samples) based on the assumption that if the 95% confidence interval (CI) excludes 0, the mediation is considered significant (Preacher et al., 2010). Third, multi-group analyses in SEM were made to evaluate if the overall model differed along gender lines, in which the critical ratio for difference (CRD) was applied to compare the structural path coefficients across two groups based on the assumption that if the absolute value of the CRD is higher than 1.965, it indicates significant inter-group difference at the *p* < 0.05 level (Preacher and Hayes, 2008).

## Results

### Descriptive Statistics and Correlation Analyses

The descriptive statistical results of the socio-demographic covariates herein are shown in Table 1. Among the 1,007 sampled young migrants, most were male and married aged 34 years on average. A majority of them had finished middle school, and only a few were illiterate. Their personal annual income fell in the range of 20,000–39,999 CNY. The mean, standard deviation, and correlation matrix of each key variable are in Table 2. The social integration, social mentality, and health status of young migrants were significantly positively correlated, that is, significant correlations existed among the key variables in this study, satisfying the prerequisites for the mediation effect test (Fang et al., 2012).

**Table 1:** Descriptive statistics (*N* = 1,007).

Variables	Category	Frequency	%
Gender	Male	540	53.62
	Female	467	46.38
Marital status	Single	200	19.86
	Married	781	77.56
	Divorced	26	2.58
Education	Illiterate	12	1.19
	Elementary school	101	10.03
	Middle school	273	27.11
	High school	210	20.85
	Associate college	160	15.89
	Bachelor	224	22.24
	Master	27	2.68
Income (personal annual income) (CNY)	<20,000	255	25.32
	20,000–39,999	350	34.76
	40,000–59,999	197	19.56
	60,000–79,999	82	8.14
	≥80,000	123	12.21
Age		Mean = 33.83	SD = 6.79

**Table 2:** Correlation analyses among key variables.

	*M*	SD	1	2	3
1. Social integration	13.146	5.080	1		
2. Social mentality	11.909	2.856	0.224^***^	1	
3. Health status	7.773	2.135	0.384^***^	0.367^***^	1

*** *p* < 0.001.

### Measurement Model

Confirmatory factor analysis was used to evaluate whether the measurement model fit the data in an adequate manner. Three latent variables were included in the model: social integration, social mentality, and health status. The assessment results showed a good model–data fit [i.e., chi-square = 60.014 (*p* < 0.001; df = 24), CFI = 0.989, and RMSEA = 0.039]: all factor loadings of the indicators of latent variables were significant at the *p* < 0.001 level; in particular, the factor loadings for social integration, social mentality, and health status ranged from 0.537 to 0.851, 0.660 to 0.807, and 0.762 to 0.827, respectively; the CR values of the latent variables were 0.826, 0.784, and 0.774 (all above 0.6), and the AVE values were 0.550, 0.549, and 0.632 (all above 0.5), indicating that the convergent validity of the model was acceptable. Furthermore, the multi-group analyses suggested the invariance of the model across the two groups at the configural, metric, and scalar levels.

### Structural Model

**Figure 2:**
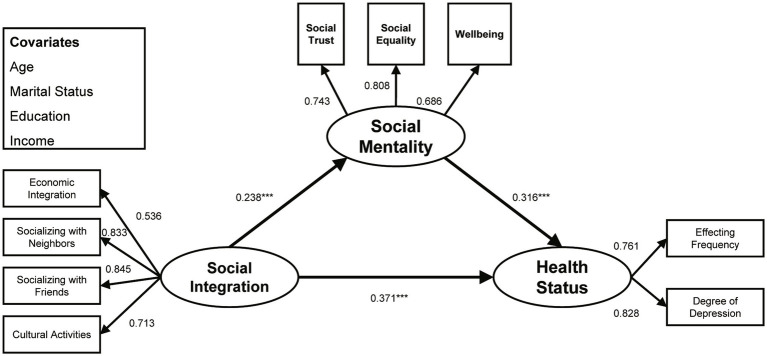
Standardized structural model (full sample). ^***^p < 0.001.

The fit indices were chi-square = 186.094, df = 54, *p* < 0.001, CFI = 0.968, TLI = 0.946, and RMSEA = 0.049, indicating a good fit with the sample data. Figure 2 and Table 3 show the full-sample model, where the action of social integration on the health of young migrants was significantly positive (*β* = 0.371, *p* < 0.001). That is to say, the higher the degree of social integration, the more robust the health. H1 was thus validated. Social mentality proved to be an important mediator in associating the social integration with the health of young migrants. Specifically, improved social integration enabled young migrants to obtain better social mentality (*β* = 0.238, *p* < 0.001), hence improved health (*β* = 0.316, *p* < 0.001), which validated H2.

**Table 3:** Results of structural model for full sample and subsamples.

Model paths			Full sample				Male sub-sample				Female sub-sample			
			B	*β*	SE	CR	B	*β*	SE	CR	B	*β*	SE	CR
Social mentality	<––	Social integration	0.153^***^	0.238	0.025	6.179	0.199^***^	0.300	0.033	6.063	0.090^*^	0.150	0.036	2.541
Health status	<––	Social mentality	0.412^***^	0.316	0.052	7.847	0.510^***^	0.392	0.068	7.523	0.257^***^	0.193	0.077	3.329
Health status	<––	Social integration	0.311^***^	0.371	0.033	9.484	0.427^***^	0.495	0.042	10.111	0.156^***^	0.194	0.046	3.377
Health status	<––	Age	0.001	0.010	0.004	0.263	0.004	0.039	0.006	0.726	−0.003	−0.034	0.006	−0.592
Health status	<––	Married	0.043	0.025	0.071	0.602	−0.013	−0.008	0.096	−0.139	0.062	0.037	0.104	0.594
Health status	<––	Divorced	0.004	0.001	0.164	0.023	0.041	0.008	0.238	0.174	−0.035	−0.009	0.219	−0.160
Health status	<––	Education	0.028^***^	0.150	0.007	3.989	0.033^**^	0.163	0.010	3.212	0.019^*^	0.112	0.009	1.995
Health status	<––	Income	0.092^***^	0.166	0.021	4.409	0.133^***^	0.232	0.029	4.622	0.071^*^	0.134	0.030	2.332

B, unstandardized coefficient; *β*, standardized coefficient; SE, standard error; CR, critical ratio.

*** *p* < 0.001;

** *p* < 0.01;

* *p* < 0.05.

Among the covariates, the more education a young migrant had, the better their health (*β* = 0.150, *p* < 0.001), and higher income levels predicted more robust health (*β* = 0.166, *p* < 0.001). However, age and marital status exerted no significant predictive effect on the health status of young migrants. Overall, the full-sample model explained 14.7% of the variance in social mentality and 41.0% of the variance in health status. The bootstrapping results are shown in Table 4. Social mentality acted as a mediator in the action of social integration on the health of young migrants (*β* = 0.075, 95% bootstrap CI [0.046, 0.112]), accounting for 16.8% of the total effect (*β* = 0.446, 95% bootstrap CI [0.378, 0.512]). The bootstrap 95% confidence intervals of the indirect effect excluded 0 to indicate significance of the indirect effect.

**Table 4:** Direct and indirect effects and 95% confidence intervals (CI).

Model pathways	Full sample			Male sub-sample			Female sub-sample		
	*β*	95% CI		*β*	95% CI		*β*	95% CI	
		Lower	Upper		Lower	Upper		Lower	Upper
*Total effect*									
Social integration → health status	0.446	0.378	0.512	0.613	0.546	0.679	0.223	0.099	0.346
*Direct effect*									
Social integration → health status	0.371	0.298	0.440	0.495	0.406	0.580	0.194	0.068	0.318
*Indirect effect*									
Social integration → social mentality → health status	0.075	0.046	0.112	0.118	0.072	0.179	0.029	0.005	0.076

*β*, standardized coefficient.

### Group Difference Tests

The multi-group analyses in SEM were done to test if the path coefficients were significantly different across genders. First, the invariance of the measurement model was tested to show that the model was invariant (*p* > 0.05). That is, its factor loadings were equal across genders. Secondly, the unconstrained structural model where the structural paths changed with gender was set against the constrained one which equalized the factor loadings, covariances, weights, and residuals between males and females. The results suggested that the unconstrained model (*χ*^2^ = 234.230, df = 108) was significantly different (*p* < 0.001) from the constrained model (*χ*^2^ = 384.398, df = 159).

**Figure 3:**
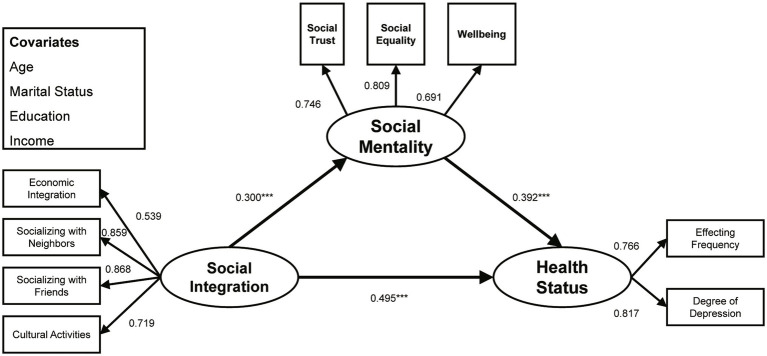
Standardized structural model (male sub-sample). ^***^p < 0.001.

**Figure 4:**
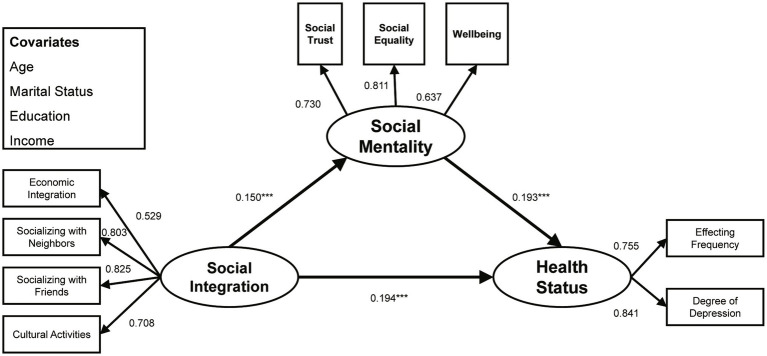
Standardized structural model (female sub-sample). ^***^p < 0.001.

The CRD tests found that the structural paths from social integration to health status (CRD = −4.480, *p* < 0.001), from social integration to social mentality (CRD = −2.273, *p* < 0.05), and from social mentality to health status (CRD = −2.519, *p* < 0.05) were significantly different along gender lines. As shown in Figures 3, 4, and Table 3, the three structural paths significantly positively affected both groups, but the degree of effect was different. Specifically, on the path from social integration to health, young male migrants were more affected (*β* = 0.495, *p* < 0.001) than young female migrants (*β* = 0.194, *p* < 0.001). On the path connecting social integration to social mentality, the influence on young male migrants (*β* = 0.300, *p* < 0.001) exceeded that of young female migrants (*β* = 0.150, *p* < 0.05). On the path connecting social mentality to health, the influence on young male migrants (*β* = 0.392, *p* < 0.001) also exceeded that of young female migrants (*β* = 0.193, *p* < 0.05).

## Discussion

This study analyzed the linkage among the social integration, social mentality, and health status of young migrants. It was found that social integration could not only directly predict the health status of young migrants, but also influence the health of young migrants through the mediating effect of social mentality. Although existing research has revealed how social integration and health are connected among young migrants (Xiong et al., 2021), this study sheds light on the psychosocial mechanism that relates social integration with health among young migrants and also demonstrates that the mechanism of social integration acting on the health of young migrants through social mentality differs significantly by gender.

In this study, increased social integration effectively improved the health status of young migrants, which is a finding that supported H1 and kept with the results of previous research. As the main force of China’s floating population, young migrants see the degree of social integration directly affect their health status (Fakhoury et al., 2021). In terms of economic integration, young migrants do not have the same access to social security and medical services as locals, and difficulties, such as the reimbursement of medical bills from places other than the registered place of residence under the New Rural Cooperative Medical Scheme, have affected their access to medical treatment (Zheng et al., 2020). In terms of acculturation, as revealed in the acculturation theory, the life changes that trigger acculturation are sometimes beneficial and offer new development opportunities to the acculturated person, but sometimes they bring about a great deal of pressure, which has a pronounced effect on the health of migrating or floating populations (Fox et al., 2017). In terms of social adaptation, young migrants have lost their original interpersonal networks, but the construction of new social networks and social interactions can lower the negative impact that social adaptation produces on their mental health and improve their social position and development of social functions (Chiang et al., 2021).

Moreover, it was found that young migrants with a high extent of social integration had a better social mentality, thereby improving their health status, which supported H2. This finding offers a new framework to explain the relationships that exist between the social integration, social mentality, and health of young migrants. Social mentality is dynamic in nature (Durkheim, 1964). China’s population mobility coincides with a transformation in the urban–rural social structure, and social mentality can reflect the changing social structure and social life of young migrants (Li and Zhao, 2018). A higher degree of social integration provides young migrants with new opportunities for participation and life resources, improves their sense of belonging and adaptability to changes in the social environment (Brailovskaia et al., 2019), and enhances their social mentality, including their social trust, sense of social equality, and subjective wellbeing, thereby buffering the effect of depression and anxiety caused by mobility pressure and effectively improving their psychological and physical health (Alfieri et al., 2019).

The multi-group analyses that focused on gender provided valuable findings. The social mentality-mediated action of social integration on the health of young migrants differed by gender, which supported H3. On the three paths (i.e., social integration affecting health, social integration affecting social mentality, and social mentality affecting health), young male migrants were more affected than young female migrants. According to previous research, the level of social integration among young female migrants is lower than that of males, which is well explained by the low employment rate of females and the fact that they assume more family responsibilities, such as caring for children and the elderly (Okumura et al., 2021). Chinese social culture has long been influenced by the traditional belief of “men outside home, women inside.” Traditional values, mores, and customs permeate daily life and convey a society’s positioning and expectations for women’s family roles through informal systems. These invisible social expectations and the belief that men are superior to women unconsciously suppress the career pursuits of women (Song and Feng, 2019). In China, young female migrants assume more responsibilities when it comes to taking care of their families and children, and their employment rate is low, making it difficult for them to truly integrate into the inflow areas (Fan and Li, 2020). Therefore, compared with women, young male migrants’ social integration level is higher, and their health status and social mentality are more easily affected by their degree of social integration (Morawa et al., 2020). Additionally, social mentalities, such as social trust and a sense of social equality (which are both closely associated with the extent of social integration), exerts a higher impact on the health of men than women (Smith et al., 2018).

## Conclusion

With a sample of young Chinese migrants, this study revealed the mechanisms of interaction that exist between social integration, social mentality, and health status within this group and verified the gender differences that emerge in these relationships. The results showed that social integration significantly positively affected the health of young migrants; that social mentality mediated the action of social integration on the health for young migrants; and that young male migrants were more susceptible to the aforementioned mechanisms than young female migrants.

Objectively speaking, some gaps exist in the study, which needs to be filled in future research. First of all, although our study combined theory and multi-group analyses in SEM to investigate the interplay among the social integration, social mentality, and health of young migrants, due to the use of pooled cross-sectional data, further longitudinal studies are warranted to deepen the causal relationships. For example, tracking data can be used to explore the interplay and development trend of the three variables and test the longitudinal mediating effect of social mentality therein (Ju and Lee, 2018). Second, in addition to social mentality, other variables may also mediate the linkage between social integration and health among young migrants, such as social capital and social status (Chen et al., 2020a), whose mediating roles can be discussed in future research. Third, due to data limitations, the measurement of the sample’s health status lacked objective physical indicators. Although self-rated health is a well-rounded indicator that effectively indicates individual health status and has proven to have good reliability and validity in the Chinese social environment (Qi and Liu, 2021), it is subject to individual cognitive biases, such as “feeling too good about oneself” and response errors. Therefore, future research may collect data on both self-rated health and objective physical health to complement the research data and draw more reliable conclusions. Finally, in the context of COVID-19 pandemic, in addition to the spread of the virus, the triggered social response, such as the community “grid” management, has a profound impact on the social integration, social mentality, and health of young migrants. Limited by data, in-depth analysis cannot be carried out for the time being. In the follow-up research, the impact of the pandemic on the health of young migrants will be discussed combined with first-hand survey data (He et al., 2020).

Although this study has certain limitations, it raises some interesting questions about young migrants in China. They have participated in creating China’s economic miracle in the process of rapid industrialization and urbanization, but can hardly get rid of the reality of being marginalized. Compared with other groups, their health status is more fragile (Deng and Law, 2020). The outbreak of the new crown epidemic in late December 2019 further highlighted the importance of targeted protection of the health and rights of the floating population. Therefore, this study offers important theoretical reference and practical guidance for improving policies related to the health of young migrants. The following are our proposals combined with the results of data analysis. First, the household registration system can be further reformed in order to build an urban–rural integrated labor market and social security system, thereby improving the economic integration of young migrants. Second, employers should be required to eliminate internal discriminatory regulations on female employment and give women equal employment status in the labor market. For example, an anti-employment discrimination law can be established to clearly define the identification of women’s employment discrimination, and punitive measures for employers’ discriminatory practice can be formulated (Wang, 2019). Third, community resources should be put into full use. For instance, more community activities can be organized and the mediation mechanism for community conflicts and disputes can be improved to promote the social adaptation and cultural integration of young migrants, helping them form good social relations with local residents and enhance their social trust, sense of security and happiness (García-Cid et al., 2020). Moreover, in the case of routine management for the new crown epidemic, professional psychological counseling can be offered to the young migrants in the community to alleviate the psychological impact of the epidemic on them, adding to the all-around support for their social integration (Li et al., 2021). Finally, social organizations, new media, and other means can be applied to promote health education, including healthy lifestyles, hygiene living habits, and medical information, in places where the floating population is relatively concentrated, so as to effectively improve the health literacy and the ability to rationally use medical services among young migrants (Marmot et al., 2008).

## Data Availability Statement

Publicly available datasets were analyzed in this study. This data can be found at: http://cgss.ruc.edu.cn/lxwm.htm.

## Author Contributions

JZho and LZ designed the model and the research framework and wrote the manuscript. JZha contributed to the data preparation and revision of the literature review. All authors contributed to the article and approved the submitted version.

## Funding

This work was sponsored by the Social Science Fund of Jiangsu Province under grant nos. 20SHC002 and 18GLD015.

## Conflict of Interest

The authors declare that the research was conducted in the absence of any commercial or financial relationships that could be construed as a potential conflict of interest.

## Publisher’s Note

All claims expressed in this article are solely those of the authors and do not necessarily represent those of their affiliated organizations, or those of the publisher, the editors and the reviewers. Any product that may be evaluated in this article, or claim that may be made by its manufacturer, is not guaranteed or endorsed by the publisher.
